# Annular closure device for disc herniation: meta-analysis of clinical outcome and complications

**DOI:** 10.1186/s12891-018-2213-5

**Published:** 2018-08-16

**Authors:** Wen Jie Choy, Kevin Phan, Ashish D. Diwan, Chon Sum Ong, Ralph J. Mobbs

**Affiliations:** 1 Faculty of Medicine, University of New South Wales (UNSW), Sydney, Australia; 2 NeuroSpine Surgery Research Group (NSURG), Prince of Wales Private Hospital, Sydney, Australia; 3 Department of Neurosurgery, Prince of Wales Private Hospital, Sydney, Australia; 4 Spine Service, Department of Orthopaedic Surgery, St. George & Sutherland Clinical School, University of New South Wales, Kogarah, 2217 New South Wales Australia; 5 Newcastle University Medicine Malaysia (NUMed), Johor, Malaysia

**Keywords:** Annular closure device, Annular repair, Recurrent disc herniation, Microdiscectomy, Barricaid, Anulex, Xclose, Lumbar intervertebral disc, Disc herniation, Meta-analysis

## Abstract

**Background:**

Lumbar intervertebral disc herniation is a common cause of lower back and leg pain, with surgical intervention (e.g. discectomy to remove the herniated disc) recommended after an appropriate period of conservative management, however the existing or increased breach of the annulus fibrosus persists with the potential of reherniation. Several prosthesis and techniques to reduce re-herniation have been proposed including implantation of an annular closure device (ACD) – Barricaid™ and an annular tissue repair system (AR) – Anulex-Xclose™. The aim of this meta-analysis is to assist surgeons determine a potential approach to reduce incidences of recurrent lumbar disc herniation and assess the current devices regarding their outcomes and complications.

**Methods:**

Four electronic full-text databases were systematically searched through September 2017. Data including outcomes of annular closure device/annular repair were extracted. All results were pooled utilising meta-analysis with weighted mean difference and odds ratio as summary statistics.

**Results:**

Four studies met inclusion criteria. Three studies reported the use of Barricaid (ACD) while one study reported the use of Anulex (AR). A total of 24 symptomatic reherniation were reported among 811 discectomies with ACD/AR as compared to 51 out of 645 in the control group (OR: 0.34; 95% CI: 0.20,0.56; I^2^ = 0%; *P* < 0.0001). Durotomies were lower among the ACD/AR patients with only 3 reported cases compared to 7 in the control group (OR: 0.54; 95% CI: 0.13, 2.23; I^2^ = 11%; *P* = 0.39). Similar outcomes for post-operative Oswestry Disability Index and visual analogue scale were obtained when both groups were compared.

**Conclusion:**

Early results showed the use of Barricaid and Anulex devices are beneficial for short term outcomes demonstrating reduction in symptomatic disc reherniation with low post-operative complication rates. Long-term studies are required to further investigate the efficacy of such devices.

## Background

Situated between vertebral bodies of the spine, the intervertebral discs (IVDs) or discs are important in maintaining a deformable space between each vertebra, assisting in flexibility and playing a role in shock absorption simultaneously [1]. Three structures make up the IVD: centrally the nucleus pulposus (NP) is surrounded by a ring of annulus fibrosus (AF) and sandwiched between two cartilaginous endplates (CEP) superiorly and inferiorly [1, 2]. A common cause of lower back and leg pain, is lumbar disc herniation (LDH) [3]. When simple measures fail to resolve patient symptoms a discectomy using various surgical approaches (e.g. open discectomy, endoscopic and microdiscectomy) are used to remove the herniated IVD and decompress the symptomatic nerve [4, 5].

In randomised controlled studies, patients with LDH who undergo discectomy have been shown to have significantly better outcomes compared to those managed conservatively [6–8]. However, 48% of LDH patients in a large spinal registry, responding to an outcome question at 1 year after surgery expressed unhappiness with the level of pain [9]. Further, 7-year survivorship analysis of large administrative databases indicates that 18% of patients undergo either a revision discectomy or spinal reconstruction following a discectomy [10]. Most likely, nerve root decompression and removal of the symptomatic disc sequestration with microdiscectomy may further weaken the AF, and exacerbate progressive dehydration of the NP which may lead to further loss of disc height. The weakened AF may also result in potential reherniation in about 0.5–25% of cases [11–14] and the loss of disc height may cause further nerve compression and radiculopathy, either in the short or long term post-surgery [15]. Such consequences can result in worsening pain which may subject the patient to seek additional surgeries, for instance fusion (i.e.: ALIF, PLIF, etc.) or artificial disc replacement, with fibrosis and inevitable consequences of the previous surgeries [15–21]. Currently, there are no definite guidelines or studies that recommend a certain approach or preventative measure towards recurrent LDH (RDH).

Symptomatic RDH is associated with higher hospital and surgical costs, repeated recovery and rehabilitation expenses, delayed return to work, and poorer outcomes as compared with the primary intervention. Expenses incurred include diagnostic and imaging testing, healthcare visits, epidural steroid injections, and revision surgeries with an estimated cost of $39,836 to $49,431 per patient [18, 22]. In addition to healthcare costs, RDH is associated with recurrent back and leg pain, affected function, loss of work days and quality of life, and increased narcotic usage and dependency [18, 23].

Various measures have been trialled to prevent RDH including aggressive removal of the NP, packing the IVD space with cellulose and other materials post-tissue-removal, sequestrectomy and fusion, but were associated with variable outcomes [24–27]. Recently, several prosthesis and techniques have been proposed to prevent the incidence of RDH which include implantation of an annular closure device (ACD) – Barricaid™ (Intrinsic Therapeutics, Inc., Woburn, MA, USA), and the use of an annular tissue repair system (AR) – Anulex-Xclose (Anulex Technologies, Minnetonka, MN) [28–31]. The Barricaid device comprises of a titanium anchor portion which is implanted into the adjacent vertebral body and a polymer mesh potion that is inserted into the affected disc, blocking the defect opening with the expectation of reducing the chance of reherniation from the same defect [30–32]; whereas the Anulex device comprises of tension band(s) each with 2 tissue anchors placed on either side of the annular defect / annular incision on the IVD to repair the defect opening in a single band or multiple band pattern which in theory repairs the defect [28]. In order to evaluate the clinical outcomes of each intervention, we conducted a meta-analysis based on the available studies to assist surgeons in evaluating the available literature, and therefore a potential approach to reduce the incidences of RDH.

## Methods

### Literature search strategy

Literature search was carried out based on PRISMA guidelines [33] and recommendations [34]. Electronic databases used include Ovid Medline, Embase, Web of Science and PubMed. To achieve the highest possible sensitivity, the search terms used were a combination of “annular device”, “annular repair”, “annulus device”, “disc herniation” and “recurrent disc herniation”. The search was performed on 13th September 2017. Further review of the reference list of all related articles was performed to identify potential studies. All relevant articles were assessed systematically utilising the inclusion and exclusion criteria.

### Selection criteria

Eligible articles for this systematic review and meta-analysis include: (1) articles discussing ACD or alternative methods to reduce rate of re-herniation, (2) articles that provide a comparison study between a population that underwent the additional procedure compared to a control group and (3) articles that provide data regarding re-herniation rates. When articles that reported the same study population were identified, those which provide the most complete data set were used. Abstracts, case reports/series and conference presentations were excluded. There were no review articles that matched our study criteria.

### Data extraction and critical appraisal

All data (text, figures and tables) were extracted from available full text reports utilising a standard proforma. Data extracted from the articles include: (1) study characteristics which covers study period, institution and country of study, average length of follow up, study size and vertebra level involved; (2) patients’ baseline traits covering age, weight or BMI and gender; (3) mean pre- and post-operation Oswestry Disability Index (ODI); (4) mean pre- and post-operation visual analogue scale (VAS) for back and legs; (5) outcome of surgery focusing on symptomatic disc re-herniation; and (6) post-operation complications including durotomy, wound complication and epidural hematoma. Estimated data from graphs were used for studies which did not report the exact mean and standard deviation for post-operative ODI or VAS. The articles were appraised according to the Dutch Cochrane Centre critical review checklist proposed by MOOSE [35].

### Statistical analysis

The weighted mean difference (WMD) and odds ratio (OR) were used as summary statistics. Both fixed- and random-effect models were tested. In the fixed-effects model, it was assumed that treatment effect in each study was the same, whereas in a random-effects model, it was assumed that there were variations between studies. χ^2^ tests were used to study heterogeneity between trials. I^2^ statistic was used to estimate the percentage of total variation across studies, owing to heterogeneity rather than chance, with values greater than 50% considered as substantial heterogeneity. I^2^ can be calculated as: I^2^ = 100% × (Q – df)/Q, with Q defined as Cochrane’s heterogeneity statistics and df defined as degree of freedom. In the present meta-analysis, the results using the random-effects model were presented to take into account the possible clinical diversity and methodological variation between studies. Specific analyses considering confounding factors were not possible because raw data were not available. All *P* values were 2-sided. All statistical analysis was conducted with Review Manager Version 5.3.2 (Cochrane Collaboration, Software Update, Oxford, United Kingdom).

Pooled analyses were portrayed via forest plots for rates of symptomatic re-herniations, durotomies and wound complications; while meta-regression was used for ODI and VAS back and leg pain.

## Results

**Fig. 1:**
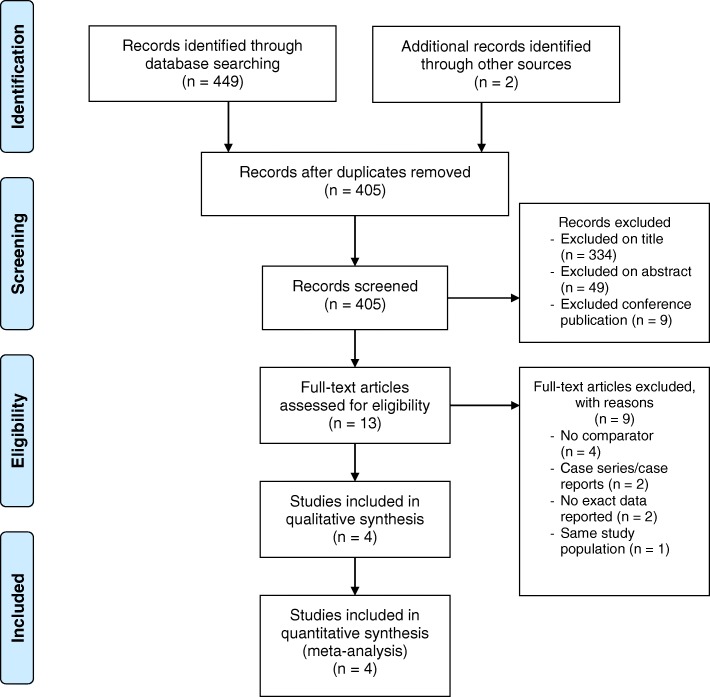
PRISMA Flow Diagram for Systematic Review and Meta-Analysis [33]

A total of 405 references were identified. Four studies [28, 30, 36, 37] met the inclusion criteria and were selected for analysis (Fig. 1). A summary of the study characteristics is shown in Table 1. The included studies were assessed for their quality and a summary is provided in Table 2. Eight hundred eleven patients underwent discectomy with an ACD or Annular Repair (ACD/AR) in these 4 studies compared to 645 patients who underwent discectomy only. All 4 studies were prospective studies with 2 of these being randomised controlled trials [28, 36]; whilst the other 2 being non-randomised comparative cohort studies [30, 37].

**Table 1:** Summary of study characteristics

Reference:	Institution	Country	Study type	Lumbar Discectomy		Average Follow up
				With ACD/AR	Without ACD/AR	
Bailey et al. [28]	34 Medical Centres	United States	P, R	478	249	24 months
Klassen et al. [36]	21 Medical Centres	Europe	P, R	272	278	90 days
Parker et al. [30]	2 Universities Medical Institution	United States	P, NR	31	46	24 months
Vukas et al. [37]	Dubrava University Hospital and Rijeka University Hospital Centre	Croatia	P, NR	30	72	24 months

*P* prospective, *R* randomised, *NR* non-randomised

**Table 2:** Quality Assessment of the Included Studies

	Bailey et al.	Klassen et al.	Parker et al.	Vukas et al.
Clear definition of study population	Yes	Yes	Yes	Yes
Clear description of outcomes and outcome assessments	Yes	Yes	Yes	Yes
Independent assessment of outcome parameters	No^a^	No^a^	No^a^	No^a^
Sufficient follow-up duration	Yes	No^b^	Yes	Yes
No selective loss of follow-up	No^c^	No^c^	Yes	Yes
Identification of confounders and prognostic factors	Yes	Yes	No^d^	No^d^

^a^, lack of blinding; ^b^, 90 days ^c^, patients fail to attend follow-up; ^d^, limitations poorly reported

### Patient characteristics

Overall, the age range of the patients were between 18 and 70 years. The reported mean age among patients who received an ACD/AR was 41.76 (range 39.92–43.60) years in 3 reported studies compared to 42.52 (range 40.72–44.31) years in the control group [28, 30, 36]. Baseline characteristics such as gender, weight, height, smoking status, diabetes or other co-morbidities were not adequately reported with only 3 studies reporting patients’ gender [28, 36, 37] and 2 reporting their BMI [28, 36].

### Clinical outcomes

**Fig. 2:**
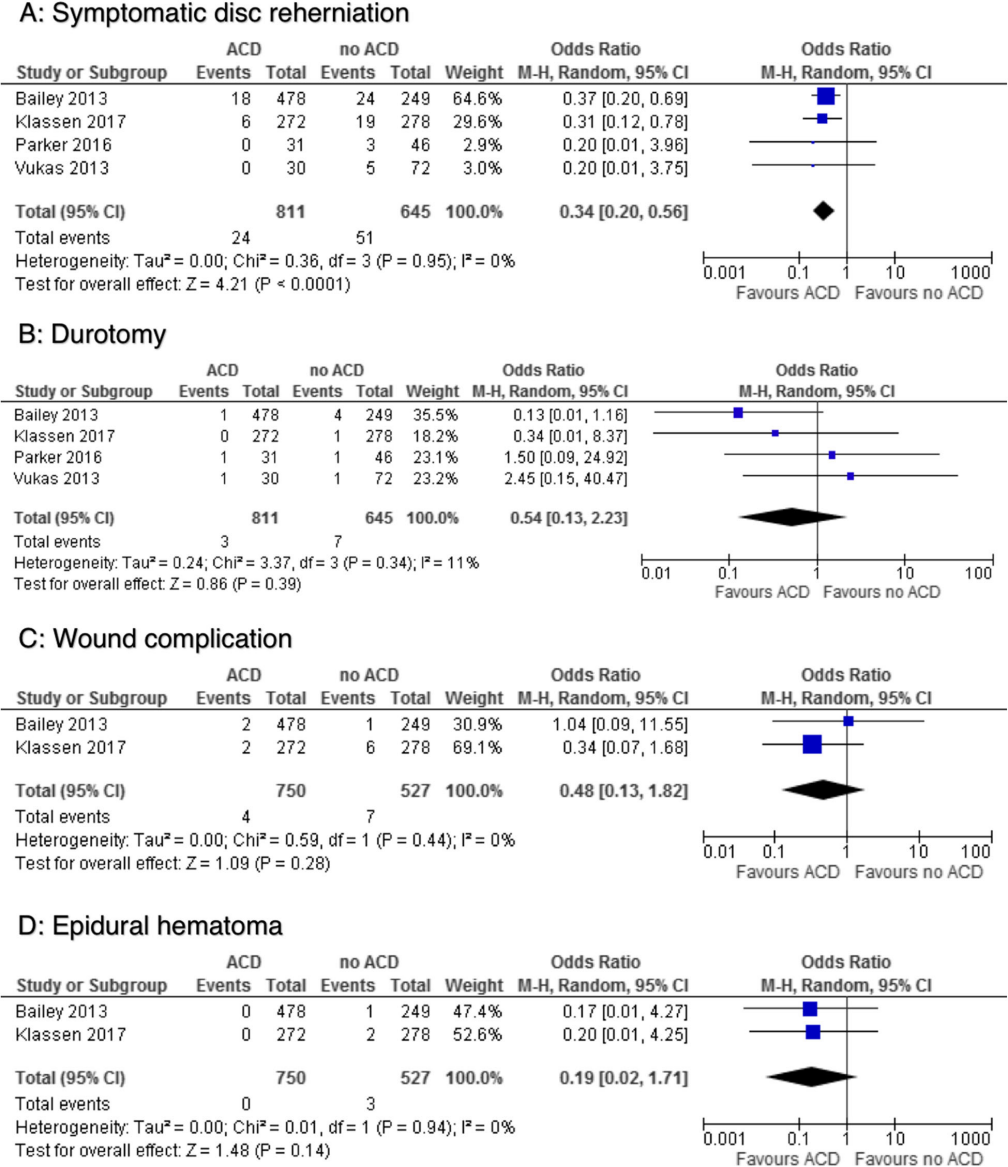
Comparison between ACD/AR group to no ACD/AR group. **a** Symptomatic disc reherniation; **b** Durotomy; **c** Wound complication; **d** Epidural hematoma

Out of the total 811 lumbar discectomies with ACD/AR, there were 24 reported symptomatic disc reherniation as compared to 51 out of 645 incidences of symptomatic reherniation among the control group (OR: 0.34; 95% CI: 0.20,0.56; I^2^ = 0%; *P* < 0.0001) (Fig. 2a). Incidence of durotomy was 3 out of 811 in the ACD/AR cohort compared to 7 out of 645 in the control group (OR:0.54; 95% CI: 0.13, 2.23; I^2^ = 11%; *P* = 0.39) (Fig. 2b). Two studies reported 4 incidences of wound complication in 750 of the ACD/AR group compared to 7 out of 527 in the control group whereas no patients had an epidural hematoma in the ACD/AR group compared to 3 out of 527 having an incidence of post-operative hematoma [28, 36] (Fig. 2c and d).

**Fig. 3:**
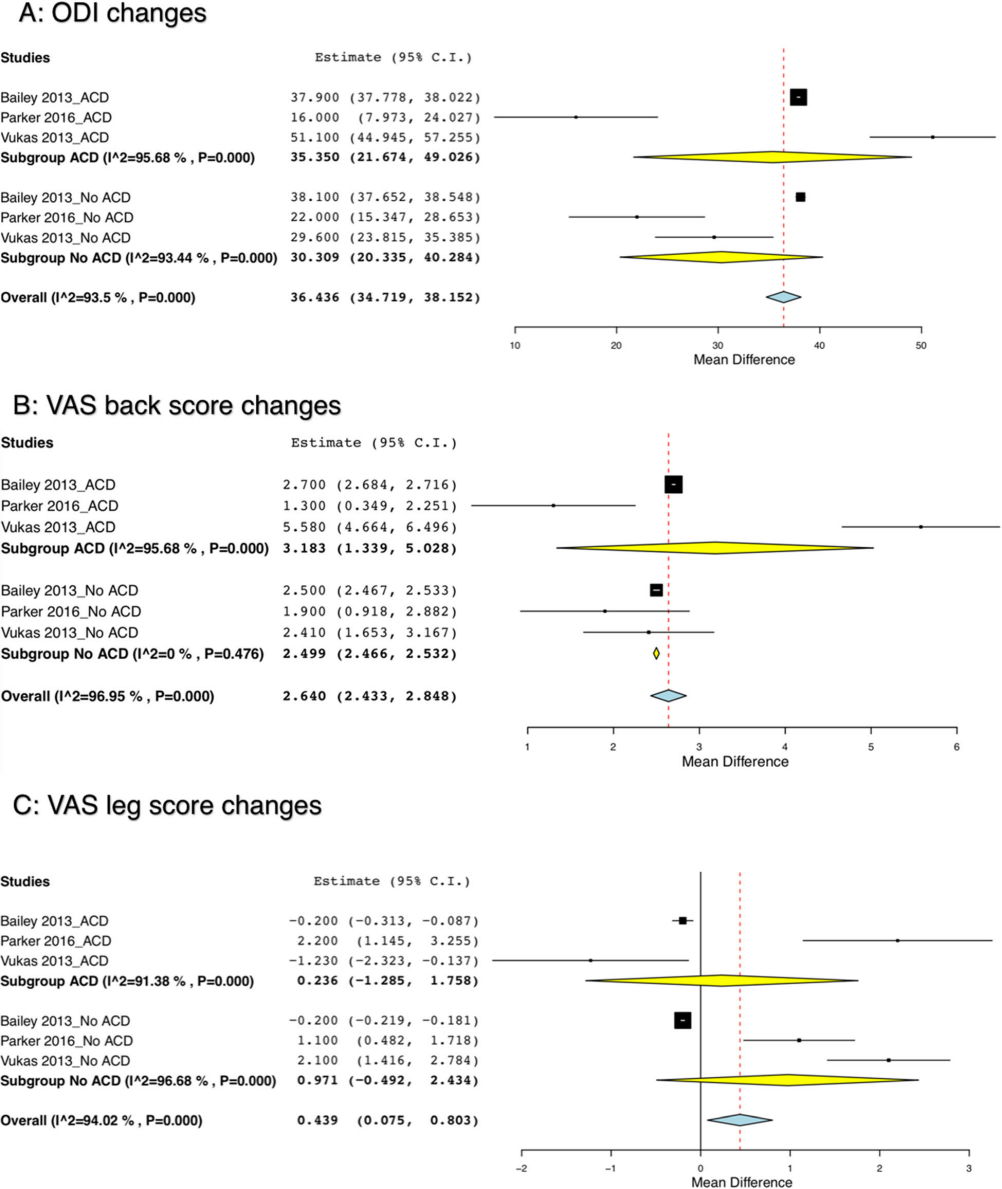
Comparison between ACD/AR group to no ACD/AR group post-intervention. **a** ODI changes; **b** VAS back score changes; **c** VAS leg score changes

Meta-regressions comparing improvements in ODI and VAS pain scores (both back and legs) for ACD/AR cohort showed similar outcomes when the ACD/AR cohort was compared to the control cohort. The results were statistically insignificant without any group being superior to the other [28, 30, 37] (Fig. 3).

## Discussion

Our results demonstrated that the use of an ACD/AR was associated with a significant reduction in symptomatic disc re-herniation [28, 30, 36, 37] compared to patients without ACD/AR, without increased risk of durotomy, wound complication or epidural hematoma [28, 36]. There was no difference in the clinical outcome scores in follow-up ODI and VAS score for both back and leg at 90 days and 2 years when the ACD/AR group is compared to the control group [28, 30, 37].

The present study is constrained by several limitations. Firstly, there is only limited data available in the literature for this new technology, with only 4 studies included for analysis. Further studies with larger sample sizes and prospective follow-up are required to confirm the presented results. The lack of available studies also resulted in shorter outcomes (90 day results) being included in our pool analysis. The lack of blinding in the studies can result in unaccounted bias. There was considerable heterogeneity in terms of ACD technology used as well as baseline characteristics, which has been shown to be an influencing factor in disc herniations. For example, A meta-analysis carried out by Huang et al. has shown statistical correlation between patients who smoke; or have disc protrusion(s); or diabetes to have an increased risk of RDH [38]. Hence future studies investigating ACD/AR among these patient population should also be carried out to evaluate the efficacy of such devices among these patients. Future randomised controlled trials (RCTs) or studies to use a similar framework of evaluation to assist more conclusive studies to be carried out in the future. Certain important aspects such as: i) patients’ baseline traits (age, weight, height and gender); ii) preoperative and postoperative ODI, VAS scores and disc height; iii) the amount of disc removed; iv) post-operative complications (durotomy and wound complications); and v) long-term symptomatic re-herniation to be included in the study. In order for proper comparison and efficacy of the ACD/AR to be evaluated, the study population should be compared to a control group as well.

While not part of the study in search for answers in support of the novel and potentially beneficial strategy we reviewed several publications that have shown that implantation of an ACD has other potential benefits apart from reducing the risk of symptomatic disc reherniation: i. Lequin et al. reported significant improvements in back and leg pain following the implantation of an ACD (Barricaid) post discectomy in 44 patients in a multicentre prospective study with one symptomatic and another asymptomatic reherniation [39]; ii. Trummer et al. carried out a prospective non-randomised trial comparing 64 ACD implantations to 137 controls and concluded that implantation of an ACD is beneficial in terms of maintaining disc space and reducing the risk of facet joint degeneration [31]; and iii. Bouma et al. reported significant reduction in symptomatic disc reherniation among their prospective non-randomised trial of 75 patients with 74 ACD implantations compared to literature [40]. Parker et al. reported that the use of ACD could potentially reduce the healthcare cost by roughly $220,000 per 100 discectomy procedures [41]. In addition, there are other case reports/series reporting similar outcomes in terms of reducing the rate of symptomatic disc reherniation and pain improvement when implantation of an ACD is used [29, 42].

However, there are limitations to the use of an ACD/AR. For instance, the Barricaid has a fixed size and has 2 parts that require implantation into both the affected IVD and the adjacent vertebral body. Should there be a significant loss of disc material, or the surface area of the herniation is too large, implantation of the Barricaid ACD would not be suitable [29, 30]. The same applies to the Anulex AR device, in which adequate disc height, and reasonable defect area will be necessary for implantation [28], hence both ARD/AR devices are only suitable to a limited group of patients. Bailey et al. proposed that 85% of patients were reported to be suitable intra-operatively for implantation of the Anulex AR device however further studies involving larger and various patient populations are needed to validate this finding [28]. Moreover, one inclusion criteria for the Barricaid ACD group was the defect has to be less than 6 mm in height and 10 mm in width [36, 37] thus making proper evaluation of the device even harder. Bouma et al. reported that only 15% of their patient population met this criterion and were eligible in their study [40]. Additionally, these devices are not suitable for patients with other spinal deformities such as spondylolisthesis as the implantation will be affected. A case report by Krukto et al. reported an incidence of aseptic instability of the ACD without signs of flora growth upon culturing as well which suggest there is still a chance of failure post-implantation [42]. Potential effects such as structural breakdown from scarring due to the surgical procedure may further weakened the surrounding structure which in turn can lead to long term poor outcomes of the ACD/AR such as implant migration.

Overall, proper efficacy of the application of ACD/AR post-discectomy could not be evaluated completely. There is only one study reporting exact data on the loss of disc height [30], in which how much disc material can be preserved with the application of these devices could not be proper gauged. Additionally, due to the limited number of RCTs carried out, we cannot conclude whether the results could be repeated. The small ACD/AR population size could also result in the small number of reported complications. Implantation of an ACD/AR will increase procedural time. Coupled with the introduction of an additional device implanted in the spine, it would undoubtedly increase the risk of durotomy or complication [28, 30, 36, 37], however this has not been borne out with the available studies to date. The results are suggestive of otherwise, in which the authors thought that the only possible explanation is surgeons are more careful when carrying out the additional procedure thus reducing post-operative complications. This is therefore a potential performance bias which can interfere with the actual data.

Our analysis leads us to the conclusion that the use of an ACD/AR is still in the early stages. There are also new methods being developed in recent times such as the application of a “Jetting Suture” technique to reduce the rate of reherniation [43]. Moreover, with recent advancement of three-dimensional printing (3DP) for patient specific implants (PSIs) in spine surgery [44–47], we believe there could be a possibility in the near future that patients with high risk of RDH be identified and receive a nucleus, or other PSI replacement to prevent RDH post-discectomy.

## Conclusion

Early results demonstrate that the use of an ACD – Barricaid and AR using Anulex post-discectomy has at least equivalent efficacy to without implantation of preventative devices without differences in pain scores and perioperative complications. Additionally, both ACD and AR are beneficial for short term outcomes (up to 2 years) for the patients demonstrating significant reduction in symptomatic disc re-herniation rates associated with low post-operative complication risk. However, given the limited amount of studies and data, we could not determine the superiority of either Barricaid or Anulex over the other. This outcome requires further studies and investigations especially with appropriate and detailed data on annular defect sizes compared with risk of recurrence. Long term follow-up is paramount to determine any potential delayed or late complications of these devices, especially the Barricaid with respects to further interventions such as fusion or disc replacement as the device may hinder operative exposure and technique. Other potential studies investigating sub groups of patients who could potentially benefit from these devices should be carried out. Future projects researching whether annular repairs will assist with regenerative technologies in maintaining mesenchymal stem cells or hydro-gel composites to stay in position post discectomy are recommended.

## Abbreviations

3DP: Three-dimensional printing
ACD: Annular closure device
AF: Annulus fibrosus
AR: Annular tissue repair system
CEP: Cartilaginous endplates
IVD: Intervertebral disc
LDH: Lumbar disc herniation
NP: Nucleus pulposus
ODI: Oswestry disability index
OR: Odds ratio
PSI: Patient specific implant
RCT: Randomised controlled trials
RDH: Recurrent lumbar disc herniation
VAS: Visual analogue scale
WMD: Weighted mean difference

## References

[1] Humzah MD, Soames RW (1988). Human intervertebral disc: structure and function. Anat Rec.

[2] Schroeder GD, Guyre CA, Vaccaro AR (2016). The epidemiology and pathophysiology of lumbar disc herniations. Seminars Spine Surg.

[3] Koes BW, van Tulder MW, Peul WC (2007). Diagnosis and treatment of sciatica. BMJ.

[4] Phan K, Dunn AE, Rao PJ, Mobbs RJ (2016). Far lateral microdiscectomy: a minimally-invasive surgical technique for the treatment of far lateral lumbar disc herniation. J Spine Surg.

[5] Yokosuka J, Oshima Y, Kaneko T, Takano Y, Inanami H, Koga H (2016). Advantages and disadvantages of posterolateral approach for percutaneous endoscopic lumbar discectomy. J Spine Surg.

[6] Weinstein JN, Lurie JD, Tosteson TD (2006). Surgical vs nonoperative treatment for lumbar disk herniation: the spine patient outcomes research trial (SPORT) observational cohort. JAMA.

[7] Lurie JD, Tosteson TD, Tosteson ANA (2014). Surgical versus non-operative treatment for lumbar disc herniation: eight-year results for the spine patient outcomes research trial (SPORT). Spine.

[8] Weinstein JN, Lurie JD, Tosteson TD (2008). Surgical versus non-operative treatment for lumbar disc herniation: four-year results for the spine patient outcomes research trial (SPORT). Spine.

[9] Fekete TF, Haschtmann D, Kleinstück FS, Porchet F, Jeszenszky D, Mannion AF (2016). What level of pain are patients happy to live with after surgery for lumbar degenerative disorders?. Spine J.

[10] Virk SS, Diwan A, Phillips FM, Sandhu H, Khan SN (2017). What is the Rate of Revision Discectomies After Primary Discectomy on a National Scale?. Clin Orthop Relat Res.

[11] Aizawa T, Ozawa H, Kusakabe T (2012). Reoperation for recurrent lumbar disc herniation: a study over a 20-year period in a Japanese population. J Orthop Sci.

[12] Berjano P, Pejrona M, Damilano M (2013). Microdiscectomy for recurrent L5–S1 disc herniation. Eur Spine J.

[13] Lebow RL, Adogwa O, Parker SL, Sharma A, Cheng J, McGirt MJ (2011). Asymptomatic same-site recurrent disc herniation after lumbar discectomy: results of a prospective longitudinal study with 2-year serial imaging. Spine (Phila Pa 1976).

[14] Swartz KR, Trost GR (2003). Recurrent lumbar disc herniation. Neurosurg Focus.

[15] Drazin D, Ugiliweneza B, Al-Khouja L (2016). Treatment of recurrent disc herniation: a systematic review. Cureus.

[16] Vialle LR, Vialle EN, Suárez Henao JE, Giraldo G (2010). LUMBAR DISC HERNIATION. Rev Bras Ortop (English Edition).

[17] Liu C, Zhou Y (2017). Percutaneous endoscopic lumbar diskectomy and minimally invasive transforaminal lumbar interbody fusion for recurrent lumbar disk herniation. World Neurosurg.

[18] Adogwa O, Parker SL, Shau DN (2012). Cost per quality-adjusted life year gained of revision neural decompression and instrumented fusion for same-level recurrent lumbar stenosis: defining the value of surgical intervention. J Neurosurg Spine.

[19] Mroz TE, Lubelski D, Williams SK (2014). Differences in the surgical treatment of recurrent lumbar disc herniation among spine surgeons in the United States. Spine J.

[20] Mobbs RJ, Phan K, Malham G, Seex K, Rao PJ (2015). Lumbar interbody fusion: techniques, indications and comparison of interbody fusion options including PLIF, TLIF, MI-TLIF, OLIF/ATP, LLIF and ALIF. J Spine Surg.

[21] Phan K, Lackey A, Chang N, et al. Anterior lumbar interbody fusion (ALIF) as an option for recurrent disc herniations: a systematic review and meta-analysis. J Spine Surg. 2017;3:587–95. 10.21037/jss.2017.11.04 PMC5760403 29354736

[22] Ambrossi GLG, McGirt MJ, Sciubba DM (2009). Recurrent lumbar disc herniation after single-level lumbar discectomy: incidence and health care cost analysis. Neurosurgery.

[23] O'Donnell JA, Anderson JT, Haas AR (2017). Treatment of recurrent lumbar disc herniation with or without fusion in workers' compensation subjects. Spine.

[24] Mastronardi L, Puzzilli F (2003). Packing of intervertebral spaces with oxidized regenerated cellulose to prevent the recurrence of lumbar disc herniation. Neurosurgery.

[25] McGirt MJ, Ambrossi GL, Datoo G (2009). Recurrent disc herniation and long-term back pain after primary lumbar discectomy: review of outcomes reported for limited versus aggressive disc removal. Neurosurgery.

[26] Thome C, Barth M, Scharf J, Schmiedek P (2005). Outcome after lumbar sequestrectomy compared with microdiscectomy: a prospective randomized study. J Neurosurg Spine.

[27] Dower A, Chatterji R, Swart A, Winder MJ (2016). Surgical management of recurrent lumbar disc herniation and the role of fusion. J. Clin. Neurosci.

[28] Bailey A, Araghi A, Blumenthal S, Huffmon GV (2013). Prospective, multicenter, randomized, controlled study of anular repair in lumbar discectomy: two-year follow-up. Spine (Phila Pa 1976).

[29] Hahn BS, Ji GY, Moon B (2014). Use of annular closure device (Barricaid(R)) for preventing lumbar disc reherniation: one-year results of three cases. Korean J Neurotrauma.

[30] Parker SL, Grahovac G, Vukas D (2016). Effect of an annular closure device (Barricaid) on same-level recurrent disk herniation and disk height loss after primary lumbar discectomy: two-year results of a multicenter prospective cohort study. Clin Spine Surg.

[31] Trummer M, Eustacchio S, Barth M, Klassen PD, Stein S (2013). Protecting facet joints post-lumbar discectomy: Barricaid annular closure device reduces risk of facet degeneration. Clin Neurol Neurosurg.

[32] Klassen PD, Hes R, Bouma GJ (2016). A multicenter, prospective, randomized study protocol to demonstrate the superiority of a bone-anchored prosthesis for anular closure used in conjunction with limited discectomy to limited discectomy alone for primary lumbar disc herniation. Int J Clin Trials.

[33] Moher D, Liberati A, Tetzlaff J, Altman DG. Preferred reporting items for systematic reviews and meta-analyses: the PRISMA statement. BMJ. 2009;339:b2535. 10.1136/bmj.b2535 PMC2714657 19622551

[34] Phan K, Mobbs RJ (2015). Systematic reviews and meta-analyses in spine surgery, neurosurgery and orthopedics: guidelines for the surgeon scientist. J Spine Surg.

[35] Stroup DF, Berlin JA, Morton SC (2000). Meta-analysis of observational studies in epidemiology: a proposal for reporting. Meta-analysis of observational studies in epidemiology (MOOSE) group. JAMA.

[36] Klassen PD, Bernstein DT, Köhler H-P (2017). Bone-anchored annular closure following lumbar discectomy reduces risk of complications and reoperations within 90 days of discharge. J Pain Res.

[37] Vukas D, Ledic D, Grahovac G, Kolic Z, Rotim K, Vilendecic M (2013). Clinical outcomes in patients after lumbar disk surgery with annular reinforcement device: two-year follow up. Acta Clin Croat.

[38] Huang W, Han Z, Liu J, Yu L, Yu X (2016). Risk factors for recurrent lumbar disc herniation: a systematic review and meta-analysis. Medicine.

[39] Lequin MB, Barth M, Thome C, Bouma GJ (2012). Primary limited lumbar discectomy with an annulus closure device: one-year clinical and radiographic results from a prospective, multi-center study. Korean J Spine.

[40] Bouma GJ, Barth M, Ledic D, Vilendecic M (2013). The high-risk discectomy patient: prevention of reherniation in patients with large anular defects using an anular closure device. Eur Spine J.

[41] Parker SL, Grahovac G, Vukas D, Ledic D, Vilendecic M, McGirt MJ (2013). Cost savings associated with prevention of recurrent lumbar disc herniation with a novel annular closure device: a multicenter prospective cohort study. J Neurol Surg A Cent Eur Neurosurg.

[42] Krutko AV, Baykov ES, Sadovoy MA (2016). Reoperation after microdiscectomy of lumbar herniation: case report. Int J Surg Case Rep.

[43] Qi L, Li M, Si H, et al. The clinical application of "jetting suture" technique in annular repair under microendoscopic discectomy: a prospective single-cohort observational study. Medicine (Baltimore). 2016;95:e4503. 10.1097/MD.0000000000004503 PMC4979855 27495101

[44] Choy WJ, Mobbs RJ, Wilcox B, Phan S, Phan K, Sutterlin CE (2017). Reconstruction of Thoracic Spine Using a Personalized 3D-Printed Vertebral Body in Adolescent with T9 Primary Bone Tumor. World Neurosurg.

[45] Mobbs RJ, Coughlan M, Thompson R, Sutterlin CE, Phan K (2017). The utility of 3D printing for surgical planning and patient-specific implant design for complex spinal pathologies: case report. J Neurosurg Spine.

[46] Phan K, Sgro A, Maharaj MM, D'Urso P, Mobbs RJ (2016). Application of a 3D custom printed patient specific spinal implant for C1/2 arthrodesis. J Spine Surg.

[47] Wilcox B, Mobbs RJ, Wu A-M, Phan K (2017). Systematic review of 3D printing in spinal surgery: the current state of play. J Spine Surg.

